# Preclinical and Clinical Evidence of Therapeutic Agents for Paclitaxel-Induced Peripheral Neuropathy

**DOI:** 10.3390/ijms22168733

**Published:** 2021-08-13

**Authors:** Takehiro Kawashiri, Mizuki Inoue, Kohei Mori, Daisuke Kobayashi, Keisuke Mine, Soichiro Ushio, Hibiki Kudamatsu, Mayako Uchida, Nobuaki Egashira, Takao Shimazoe

**Affiliations:** 1 Department of Clinical Pharmacy and Pharmaceutical Care, Graduate School of Pharmaceutical Sciences, Kyushu University, Fukuoka 812-8582, Japan; inoue.mizuki.049@s.kyushu-u.ac.jp (M.I.); yaku01177@gmail.com (K.M.); kobayashi.daisuke.223@m.kyushu-u.ac.jp (D.K.); mine.keisuke.035@s.kyushu-u.ac.jp (K.M.); kudamatsu.hibiki.555@s.kyushu-u.ac.jp (H.K.); shimazoe@phw.med.kyushu-u.ac.jp (T.S.); 2 Department of Pharmacy, Okayama University Hospital, Okayama 700-8558, Japan; s-ushio@okayama-u.ac.jp; 3 Department of Clinical Pharmacy, Faculty of Pharmaceutical Sciences, Doshisha Women’s College of Liberal Arts, Kyoto 610-0395, Japan; m-uchida@dwc.doshisha.ac.jp; 4 Department of Pharmacy, Kyushu University Hospital, Fukuoka 812-8582, Japan; egashira.nobuaki.696@m.kyushu-u.ac.jp

**Keywords:** paclitaxel, peripheral neuropathy, preclinical data, clinical evidence, adverse effects

## Abstract

Paclitaxel is an essential drug in the chemotherapy of ovarian, non-small cell lung, breast, gastric, endometrial, and pancreatic cancers. However, it frequently causes peripheral neuropathy as a dose-limiting factor. Animal models of paclitaxel-induced peripheral neuropathy (PIPN) have been established. The mechanisms of PIPN development have been elucidated, and many drugs and agents have been proven to have neuroprotective effects in basic studies. In addition, some of these drugs have been validated in clinical studies for their inhibitory PIPN effects. This review summarizes the basic and clinical evidence for therapeutic or prophylactic effects for PIPN. In pre-clinical research, many reports exist of neuropathy inhibitors that target oxidative stress, inflammatory response, ion channels, transient receptor potential (TRP) channels, cannabinoid receptors, and the monoamine nervous system. Alternatively, very few drugs have demonstrated PIPN efficacy in clinical trials. Thus, enhancing translational research to translate pre-clinical research into clinical research is important.

## 1. Introduction

Paclitaxel and albumin-bound paclitaxel are important drugs in the treatment of ovarian [1,2], non-small cell lung [3,4], breast [5,6,7], gastric [8,9], endometrial [10], and pancreatic [11] cancers. However, they cause peripheral neuropathy as an adverse event. In paclitaxel-induced peripheral neuropathy (PIPN), many patients develop sensory abnormalities (e.g., numbness, pain, and burning sensation in the hands and feet) [12]. PIPN is a dose-limiting factor that causes difficulty in continuing cancer chemotherapy [13]. However, no evidence-based prophylactic agents for PIPN were noted [14]. Since the late 1990s, many studies on the mechanism and therapeutic or prophylactic agents using PIPN animal models have been reported [15,16,17]. In addition, the mechanisms of PIPN development have been gradually clarified [18]. This study reviewed the preclinical and clinical evidence of therapeutic or prophylactic agents for PIPN.

## 2. Methods

### 2.1. Preclinical Evidence

All articles found in PubMed with the search term “paclitaxel neuropathy or paclitaxel neurotoxicity” were surveyed. The last search date was 30 April 2021. Clinical studies and reports that did not include information on therapeutic agents were excluded from the analysis. Articles referring to the effects of local rather than systemic administration and articles published before 2015 were also excluded. Information on the name and dosage of the drugs that showed statistically significant improvement, mechanism of action, and the animal species in which they were used were extracted in the surveyed papers.

### 2.2. Clinical Evidence

The articles found in PubMed with the search term “paclitaxel neuropathy or paclitaxel neurotoxicity” limited to “Randomized Controlled Trial” and “Meta-Analysis” were analyzed. The last search date was 30 April 2021. Reports other than trials about peripheral neuropathy were excluded. Moreover, information such as the investigational drug and its dosage, chemotherapy received by the patient, study design, number of patients, and results were collected.

## 3. Results

### 3.1. Therapeutic Agents in Preclinical Evidence

In PubMed, 2667 articles were found when using the search term “paclitaxel neuropathy or paclitaxel neurotoxicity”. Of these, 150 articles reported on drugs that inhibit PIPN in animal studies. The following is a summary of the drugs that had therapeutic PIPN effects in these basic studies (Table 1).

#### 3.1.1. Antioxidants and Mitochondria-Protective Agents

Many previous preclinical reports support that oxidative stress and mitochondrial dysfunction play a role in PIPN [31,104,105,106]. Vitamin C, rotenone, tempol, and curcumin which are widely known for their antioxidant effects, have been reported to alleviate PIPN in rodents [20,21,27]. Among the approved drugs, duloxetine, lacosamide, pregabalin, and rosuvastatin have also been reported to reverse PIPN via their antioxidant effects [23,28,32]. Moreover, many agents, which have antioxidant effects, inhibit PIPN in preclinical studies [19,22,24,25,26,29,30,31,33,34].

#### 3.1.2. Anti-Inflammatory Agents

Inflammatory cytokines (e.g., interleukin-1 beta (IL-1β), interleukin-6 (IL-6), and tumor necrosis factor-α (TNF-α)) and chemokines (e.g., chemokine (C-X-C motif) ligand (CXCL) family) were elevated in the peripheral sites, spinal cord, and of paclitaxel-treated animals, and many agents reduced the peripheral neuropathy symptoms via their anti-inflammatory effects [19,21,22,24,25,27,28,32,33,34,35,36,37,38,39,41,42,44,45,46,47,49,50,51,52,53,54,56]. Activations of astrocytes and microglia were also observed in the spinal dorsal horn after paclitaxel administrations, and many agents including minocycline attenuated PIPN via the inhibition of these spinal changes and prevented neurological damage [40,43,44,48].

#### 3.1.3. Ion Channel Inhibitors and Activators

Some activators of potassium channels, especially Kv7, have been shown to suppress PIPN [57,58]. Focusing on calcium channels, T-type calcium channel blockers have been reported to alleviate PIPN symptoms [54,59].

#### 3.1.4. Transient Receptor Potential (TRP) Modulators

Temperature-sensitive cation channels (e.g., transient receptor potential vanilloid 4 (TRPV4), transient receptor potential vanilloid 1 (TRPV1), and transient receptor potential ankyrin 1 (TRPA1)) have been reported to be involved in PIPN [61,107,108,109]. Many drugs have also been reported to improve PIPN by downregulating or inhibiting TRP channels [55,60,61,62,63,64].

#### 3.1.5. Cannabinoid Receptor Agonists

Many studies have shown that cannabinoid receptor agonists and related substances can suppress PIPN symptoms [48,49,56,69,70,71]. In particular, some reports exist that selective CB_2_ agonists have an ability to suppress PIPN [48,56].

#### 3.1.6. Modulators of Monoamine Nervous System

Monoamines, including noradrenaline and serotonin, play an important role in the descending pain inhibitory system [110]. Some drugs and agents (e.g., quetiapine, reboxetine, venlafaxine, and bee venom) also showed analgesic effects by modulating the monoamine nervous system in the PIPN animal models [73,74,75,76].

#### 3.1.7. Others

In addition to the aforementioned, many other drugs have been identified to reduce PIPN via several therapeutic targets, such as glutamate nerve systems [65,66], phosphodiesterase (PDE) [67,68], opioid receptors [72], acetylcholine receptor [77,78,79,80], cAMP/protein kinase A (PKA) signal [40], protein kinase C (PKC) [62,81], mitogen-activated protein kinase (MAPK) [28,39,49,53,56,81,82,83], organic anion-transporting polypeptide 1b2 (OATP1B2) [84], mammalian target of rapamycin (mTOR) [51], and others [85,86,87,88,89,90,91,92,93,94,95,96,97,98,99,100,101,102,103], at the pre-clinical research level.

### 3.2. Therapeutic Agents in Clinical Evidence

In PubMed, 1175 articles were found when using the search term “paclitaxel neuropathy or paclitaxel neurotoxicity” limited to “Randomized Controlled Trial” and “Meta-Analysis”. After excluding reports other than about PIPN, the authors found 19 reports considered to be clinically important. A summarized list of the representative randomized controlled trials and meta-analyses on prophylactic and therapeutic agents for PIPN is shown below in Table 2.

Duloxetine was tested in a randomized, double-blind, placebo-controlled, crossover trial, for its ability to treat neuropathy in patients with taxane or platinum [115]. In this study, relative risks (RRs) (95% confidence interval (95% CI)) of experiencing 30% and 50% pain reduction were 1.96 (1.15–3.35) and 2.43 (1.11–5.30), respectively. However, in a subanalysis in taxane-treated patients, RRs (95% CI) of experiencing 30% and 50% pain reduction were 0.97 (0.41–2.32) and 1.22 (0.35–4.18), respectively (not significant). Duloxetine significantly improved numbness and pain compared with vitamin B12 in a randomized, open-label, crossover study of patients who received chemotherapy including other anticancer drugs, as well as paclitaxel [114].

Pregabalin significantly improved the grade and score of taxane-related neuropathy compared with duloxetine in a randomized, double-blind, controlled study [125]. Moreover, pregabalin did not improve treatment-related pain and neuropathy scores related to paclitaxel in a randomized, double-blind, placebo-controlled, multicenter study [126]. Gabapentin was reported to significantly reduce the incidence of grade ≥2 PIPN and changes in nerve conduction velocity (NCV) in a randomized, double-blind, placebo-controlled study [122].

Omega-3 fatty acids significantly improved the incidence of peripheral neuropathy associated with paclitaxel administration in a randomized, double-blind, placebo-controlled study [122]. In a meta-analysis that included not only paclitaxel-treated patients but also oxaliplatin-treated patients, the suppressive effects of omega-3 fatty acids on neuropathy were significant [121]. Vitamin E significantly improved the incidence and scores of neuropathies in both a randomized, controlled study of patients with paclitaxel [128] and patients with paclitaxel or cisplatin [129]. Amifostine significantly improved paresthesia and sensory motor impairment in a randomized controlled study of paclitaxel/carboplatin-treated patients [112]. However, it did not significantly improve neuropathy in a randomized controlled study of paclitaxel-treated patients [113]. Additionally, minocycline, N-acetylcysteine, and eicosapentenoic acid (EPA) have been reported to improve peripheral neuropathy associated with paclitaxel [119,120,123]. Moreover, glutamate, glutathione, poly ADP-ribose polymerase (PARP) inhibitors, and human leukemia inhibitory factor (LIF) did not show any significant effect on PIPN in randomized controlled trials or meta-analyses [117,118,124,127]. Long-term administration of acetyl-L-carnitine significantly worsened taxane-related peripheral neuropathy in a randomized, double-blind, placebo-controlled, multicenter study [111].

As described above, few drugs have shown clear therapeutic PIPN effects in clinical trials. Thus, according to the clinical practice guideline updated by the American Society of Clinical Oncology in 2020, no agents have yet to be recommended for preventing chemotherapy-induced peripheral neuropathy and only duloxetine may be used as a treatment for neuropathy [14].

## 4. Discussion

The PIPN mechanism has been recently elucidated in basic studies, and many drugs and agents targeting this mechanism have been explored and identified for PIPN therapy or prophylaxis [18]. In particular, many inhibitors of neuropathy targeting oxidative stress, inflammatory response, ion channels, TRP channels, cannabinoid receptor, and monoamine nervous system have been identified as candidates for inhibiting PIPN in animal research. In particular, more reports of inhibitors targeting peripheral and central inflammatory responses, TRP channels, and cannabinoid receptors were noted compared with pre-clinical research reports on oxaliplatin-induced peripheral neuropathy [130]. Targeting these may be useful in the search for PIPN-specific therapeutics.

Alternatively, very few drugs have shown the efficacy for PIPN in clinical trials. The American Society of Clinical Oncology’s clinical practice guideline states that only duloxetine can be used for the treatment of chemotherapy-induced peripheral neuropathy [14]. In a randomized double-blind placebo-controlled crossover study, duloxetine has been reported to improve neuropathic pain caused by taxanes and platinum [115]. However, a subanalysis of that study also showed a weak inhibitory effect of duloxetine on taxanes in neuropathic pain [115]. Thus, few evidence-based treatments for PIPN were noted.

Most clinical studies examined the preventive rather than curative effects on PIPN. Meanwhile, pre-clinical studies have explored many therapeutic targets for PIPN. Of these, agents on the therapeutic targets that inhibit pain or sensory abnormalities, such as K channel, Ca channel, TRP channels, glutamate, cannabinoid receptors, opioid receptors, and monoamine nervous system, may have curative effects on PIPN that has already developed. More information on the clinical studies of these agents will make it possible to approach PIPN from both a preventive and curative perspective.

While many drugs have been reported in pre-clinical research as having the potential to inhibit the PIPN, few drugs have developed sufficient evidence in clinical studies. The valley of death between basic studies and clinical applications is caused by many issues, including the difference between clinical symptoms and animal assessment methods, the cost and time of conducting clinical research, safety considerations in clinical application, and the lack of collaboration between basic and clinical researchers. Thus, promoting translational research, that is, to bridge pre-clinical research to clinical research is important.

## Figures and Tables

**Table 1:** The therapeutic agents for paclitaxel-induced peripheral neuropathy in preclinical experiments.

Therapeutic Targets	Therapeutic Agents	Dose	Animals	Symptoms that Showed Improvement	Mechanisms	References
Oxidative stress and mitochondrial disfunction	Anakinra, IL-1β antagonist	50–100 mg/kg, i.p.	Rats	Pain threshold	Reductions of MDA, MPO and IL-1β and increase in GSH in paws	[19]
	Antimycin A	0.2–0.6 mg/kg, i.p.	Rats	Mechanical hypersensitivity	Inhibition of mitochondrial complex III	[20]
	Curcumin	100–200 mg/kg, p.o.	Rats	Histological changes in spinal cord and sciatic nerve	Reduction of NF-κB, TNF-α, IL-6, iNOS and GFAP, p53, caspase-3, Apaf-1, LC3A, LC3B and beclin-1, and increase in Nrf2, HO-1, NQO1, Bcl-2, and Bcl-xL.	[21]
	Divya-Peedantak-Kwath, a herbal decoction	69–615 mg/kg, p.o.	Mice	Thermal hyperalgesia, mechanical allodynia and hyperalgesia, and axonal degeneration	Suppression of oxidative stress and inflammation	[22]
	Duloxetine	10–30 mg/kg, i.p.	Mice	Mechanical hyperalgesia and thermal nociception	Inhibiting PARP and p53 activation and regulating Bcl-2 family to reverse oxidative stress and apoptosis	[23]
	Evodiamine	5 mg/kg	Rats	Mechanical hypersensitivity and thermal hypersensitivity	Downregulation of inflammatory and chemoattractant cytokines (IL-1β, IL-6, TNF-α, and MCP-1), oxidative stress, and mitochondrial dysfunction in DRG.	[24]
	Flavonol	25–200 mg/kg, s.c.	Mice	Tactile allodynia, cold allodynia and thermal hyperalgesia	Inhibitions of TNF-α, IL-1β and free radicals	[25]
	Ghrelin	300 nmol/kg, i.p.	Mice	Mechanical sensitivity, thermal sensitivity, DRG damage (ATF-3 positive cells), and density of IENF	Decreases in plasma oxidative and nitrosative stress and increases in UCP2, SOD2, and PGC-1α	[26]
	GKT137831, a NOX4 inhibitor	1 mg/kg, i.p.	Rats	Mechanical sensitivity and thermal sensitivity	Decreases of proinflammatory cytokines (IL-1β, IL-6, and TNF-α) in the DRG	[27]
	Lacosamide	30 mg/kg, p.o.	Rats	Thermal hyperalgesia and cold allodynia	Upregulation of total antioxidant capacity and NGF, and downregulation of NF-kB p65, TNF-α, active caspase-3, Notch1 receptor, p-p38, and IL-6/p-JAK2/p-STAT3	[28]
	Melatonin	5–50 mg/kg, p.o.	Rats	Mechanical sensitivity	Reduction of mitochondrial damage	[29]
	Nicotinamide riboside	200 mg/kg, p.o.	Rats	Tactile hypersensitivity	N.A.	[30]
	Phenyl-N-tert-butylnitrone	100 mg/kg, i.p.	Mice	Mechanical hypersensitivity	N.A.	[31]
	Pregabalin	30 mg/kg, p.o.	Rats	Thermal hyperalgesia and cold allodynia	Upregulation of total antioxidant capacity and NGF, and downregulation of NF-kB p65, TNF-α, active caspase-3, Notch1 receptor, p-p38, and IL-6/p-JAK2/p-STAT3	[28]
	Rosuvastatin	10 mg/kg, i.p.	Mice	Thermal hyperalgesia, cold hyperalgesia, and mechanical allodynia	Downregulations of IL-1β, oxidative stress	[32]
	Rotenone	1–5 mg/kg, i.p.	Rats	Mechanical hypersensitivity	Inhibition of mitochondrial complex I	[20]
	Tempol, a mimetic of SOD	20 mg/kg, i.p.	Rats	Mechanical sensitivity and thermal sensitivity	Decreases of proinflammatory cytokines such as IL-1β, IL-6 and TNF-α in the DRG	[27]
	Trimethoxy flavones	25–200 mg/kg, s.c.	Mice	Tactile allodynia, cold allodynia, and thermal hyperalgesia	Inhibitions of TNF-α, IL-1β and free radicals	[33]
	Umbelliprenin, a prenylated coumarin	12.5–25 mg/kg, i.p.	Mice	Thermal hyperalgesia	Decrease in serum IL-6 levels and oxidative stress	[34]
	Vitamin C	500 mg/kg, i.p.	Rats	Mechanical sensitivity and thermal sensitivity	Decreases of proinflammatory cytokines (IL-1β, IL-6 and TNF-α) in the DRG	[27]
Inflammatory	3-Hydroxyflavone	25–75 mg/kg, i.p.	Rats	Tactile allodynia, cold allodynia, thermal hyperalgesia, and heat-hyperalgesia	Suppressions of TNF-α, IL-1β, IL-6, CGRP, and substance P in the spinal cord, and inhibition of the receptor of substance P	[35]
	AMD3100, a CXCR4 antagonist	8 mg/kg, i.p.	Mice	Mechanical allodynia	N.A.	[36]
	Anakinra, IL-1β antagonist	50–100 mg/kg, i.p.	Rats	Pain threshold	Reductions of MDA, MPO and IL-1β and increase in GSH in paws	[19]
	Anti-HMGB1-neutralizing antibody	1 mg/kg, i.p.	Mice	Mechanical allodynia	N.A.	[36]
	Berberine	5–20 mg/kg, i.p.	Mice	Thermal hyperalgesia	N.A.	[37]
	Choline-fenofibrate	6–24 mg/kg, i.p., 15–60 mg/kg, p.o.	Mice	Mechanical hyperalgesia, cold hyperalgesia, and sensory nerve compound action potential amplitude	Regulation of PPAR-⍺ expression and decrease neuroinflammation in DRG	[38]
	Curcumin	100–200 mg/kg, p.o.	Rats	Histological changes in the spinal cord and sciatic nerve	Reductions of NF-κB, TNF-α, IL-6, iNOS and GFAP, p53, caspase-3, Apaf-1, LC3A, LC3B and beclin-1, and increase in Nrf2, HO-1, NQO1, Bcl-2, and Bcl-xL.	[21]
	Divya-Peedantak-Kwath, a herbal decoction	69–615 mg/kg, p.o.	Mice	Thermal hyperalgesia, mechanical allodynia and hyperalgesia, and axonal degeneration	Suppressions of oxidative stress and inflammation	[22]
	Duloxetine	30 mg/kg/day, i.p.	Mice	Mechanical hyperalgesia, thermal hyperalgesia, and loss of IENF	Decreases in NF-κB, p-p38, IL-6, and TNF-α in DRG	[39]
	ESI-09, a Epac inhibitor	20 mg/kg, p.o.	Mice	Mechanical allodynia and number of IENF	Suppression of spinal cord astrocyte activation	[40]
	Etanercept	2 mg/kg, i.p.	Rats	Mechanical hypersensitivity and cold hypersensitivity	Blocking of TNF-α signaling	[41]
	Evodiamine	5 mg/kg	Rats	Mechanical hypersensitivity and thermal hypersensitivity	Downregulation of inflammatory and chemoattractant cytokines (IL-1β, IL-6, TNF-α, and MCP-1), oxidative stress, and mitochondrial dysfunction in DRG.	[24]
	Fenofibrate	Diet with 0.2% or 0.4% fenofibrate	Mice	Mechanical allodynia, cold allodynia, SNAP amplitude, and intra-epidermal nerve fibers density	Regulation of PPAR-α expression and reduction in neuroinflammation	[42]
	Fenofibrate	100–150 mg/kg, i.p., 300–600 mg/kg, p.o.	Mice	Mechanical hyperalgesia, cold hyperalgesia, and sensory nerve compound action potential amplitude	Regulation of PPAR-⍺ expression and decrease neuroinflammation in DRG	[38]
	Fenofibric acid	6–24 mg/kg, i.p., 30–90 mg/kg, p.o.	Mice	Mechanical hyperalgesia, cold hyperalgesia, and sensory nerve compound action potential amplitude	Regulation of PPAR-⍺ expression and decrease neuroinflammation in DRG	[38]
	Flavonol	25–200 mg/kg, s.c.	Mice	Tactile allodynia, cold allodynia, and thermal hyperalgesia	Inhibitions of TNF-α, IL-1β and free radicals	[25]
	FPS-ZM1, a RAGE antagonist	1 mg/kg, i.p.	Mice	Mechanical allodynia	N.A.	[36]
	GKT137831, a NOX4 inhibitor	1 mg/kg, i.p.	Rats	Mechanical sensitivity and thermal sensitivity	Decreases of proinflammatory cytokines (IL-1β, IL-6, and TNF-α) in the DRG	[27]
	Human intravenous immunoglobulin	1 g/kg, i.v.	Rats	Mechanical allodynia, loss of IENF, and distal axonal degeneration	Suppression of the axonopathy with macrophage infiltration	[43]
	Icariin	100 mg/kg, p.o.	Rats	Mechanical allodynia	Downregulations of TNF-α, IL-1β, IL-6 and astrocyte activation in spinal cord via SIRT1 activation	[44]
	IL-1 receptor antagonist	3 mg/kg, i.p.	Rats	Mechanical hypersensitivity and cold hypersensitivity	N.A.	[41]
	JTC-801	0.01–0.05 mg/kg, i.v.	Rats	Mechanical allodynia	Decreases in PI3K, p-Akt, and inflammatory cytokines in the DRG	[45]
	Lacosamide	30 mg/kg, p.o.	Rats	Thermal hyperalgesia and cold allodynia	Upregulation of total antioxidant capacity and NGF, and downregulation of NF-kB p65, TNF-α, active caspase-3, Notch1 receptor, p-p38, and IL-6/p-JAK2/p-STAT3	[28]
	Losartan	20–100 mg/kg, i.p.	Rats	Mechanical hyperalgesia	Decrease in inflammatory cytokines including IL-1β and TNF-α in the DRG	[46]
	Losartan	100 mg/kg, p.o.	Rats	Mechanical allodynia	Attenuations of neuroinflammatory changes and expression of pro-resolving markers (arginase 1 and IL-10) indicating a possible shift in macrophage polarization	[47]
	Low-molecular-weight heparin, a rage antagonist	2.5 mg/kg. i.p.	Mice	Mechanical allodynia	N.A.	[36]
	LPS-R, a TLR4 antagonist	0.5 mg/kg, i.p.	Mice	Mechanical allodynia	N.A.	[36]
	MDA7, a CB₂ agonist	15 mg/kg, i.p.	Rats	Mechanical allodynia	Downregulations of IRF8, P2X4, CaMKIIα, p-CREB, FosB, BDNF, GluR1 and NR2B, and increase in the expression of K^+^-Cl^-^ cotransporter	[48]
	MJN110, a MAGL inhibitor	4–40 mg/kg, i.p.	Mice	Mechanical allodynia	Downregulations of MCP-1, CCL2 and p-p38 in DRG as well as MCP-1 in the spinal dorsal horn	[49]
	Polaprezinc	3 mg/kg, p.o.	Rats	Mechanical allodynia	Suppression of macrophage migration into DRG	[50]
	Pregabalin	30 mg/kg, p.o.	Rats	Thermal hyperalgesia and cold allodynia	Upregulation of total antioxidant capacity and NGF, and downregulation of NF-kB p65, TNF-α, active caspase-3, Notch1 receptor, p-p38, and IL-6/p-JAK2/p-STAT3	[28]
	Rapamycin	5 mg/kg, i.p.	Rats	Mechanical hypersensitivity and thermal hypersensitivity	Decreases of IL-1β, IL-6, TNF-α, substance P and CGRP in DRG.	[51]
	Reparixin	8 mg/hr/kg using micro–osmotic pumps	Rats	Mechanical allodynia and cold allodynia	Inhibition of IL-8/CXCR1/2 pathway and suppressions of p-FAK, p-JAK2/p-STAT3, and PI3K-p-cortactin activation	[52]
	Rosuvastatin	10 mg/kg, i.p.	Mice	Thermal hyperalgesia, cold hyperalgesia, and mechanical allodynia	Downregulations of IL-1β and oxidative stress	[32]
	S504393, a CCR2 antagonist	5 mg/kg, i.p.	Rats	Mechanical hypersensitivity and cold hypersensitivity	N.A.	[41]
	Siwei Jianbu decoction	5–10 g/kg, i.g.	Mice	Mechanical hyperalgesia and thermal nociception	Inhibiting the JNK, ERK1/2 phosphorylation, NF-κB, TNF-α, IL-1β, and IL-6.	[53]
	TAK242, a TLR4 antagonist	1–3 mg/kg, i.p.	Rats	Mechanical hypersensitivity	Antagonism of TLR4	[54]
	TAK242, a TLR4 antagonist	3 mg/kg, i.p.	Mice	Mechanical allodynia	N.A.	[55]
	Tempol, a mimetic of SOD	20 mg/kg, i.p.	Rats	Mechanical sensitivity and thermal sensitivity	Decreases of proinflammatory cytokines (IL-1β, IL-6 and TNF-α) in the DRG	[27]
	Thrombomodulin alfa	1–3 mg/kg, i.p.	Mice	Mechanical allodynia	N.A.	[36]
	Trimethoxy flavones	25–200 mg/kg, s.c.	Mice	Tactile allodynia, cold allodynia, and thermal hyperalgesia	Inhibitions of TNF-α, IL-1β and free radicals	[33]
	Umbelliprenin, a prenylated coumarin	12.5–25 mg/kg, i.p.	Mice	Thermal hyperalgesia	Decreases in serum IL-6 levels and oxidative stress	[34]
	Vitamin C	500 mg/kg, i.p.	Rats	Mechanical sensitivity and thermal sensitivity	Decreases of proinflammatory cytokines (IL-1β, IL-6 and TNF-α) in the DRG	[27]
	β-caryophyllene, a CB2 agonist	25 mg/kg, p.o.	Mice	Mechanical allodynia	Through CB2-activation in the CNS and posterior inhibition of p38 MAPK/NF-κB activation and cytokine release	[56]
K channel	3-Carboxyphenyl isothiocyanate	1.33–13.31 µmol/kg, s.c.	Mice	Cold hypersensitivity	Release H_2_S activating Kv7 channel	[57]
	Allyl isothiocyanate	1.33–13.31 µmol/kg, s.c.	Mice	Cold hypersensitivity	Release H_2_S activating Kv7 channel	[57]
	Phenyl isothiocyanate	4.43–13.31 µmol/kg, s.c.	Mice	Cold hypersensitivity	Release H_2_S activating Kv7 channel	[57]
	Retigabine	10 mg/kg, i.p.	Rats	Mechanical allodynia, IENF density, and morphological alteration of mitochondria in peripheral nerve	Specific KCNQ/Kv7 channel opener	[58]
	Sodium hydrosulfide hydrate	13.31–38 µmol/kg, s.c.	Mice	Cold hypersensitivity	Release H_2_S activating Kv7 channel	[57]
Ca channel	ML218, a T-type calcium channel blocker	1–10 mg/kg, i.p.	Rats	Mechanical hypersensitivity	Inhibition of Cav3.2	[54]
	RQ-00311651, a T-type calcium channel blocker	10–40 mg/kg, i.p.	Mice and rats	Mechanical hyperalgesia	Block of Cav3.1/Cav3.2 T channels	[59]
TRP channel	AMG9810	30 mg/kg, p.o.	Rats	Mechanical allodynia, hyperalgesia, and thermal hyperalgesia	TRPV1 antagonism	[60]
	Capsazepine	30 mg/kg, s.c.	Rats	Thermal hyperalgesia	TRPV1 antagonism	[61]
	HC-067047, a TRPV4 antagonist	10 mg/kg, i.p.	Mice	Mechanical hyperalgesia	TRPV4 antagonism	[62]
	Quercetin	20–60 mg/kg, i.p.	Rats and mice	Heat hyperalgesia and mechanical allodynia	Suppression of PKCε and TRPV1 in the spinal cords and DRG	[63]
	Ruthenium red	3 mg/kg, s.c.	Rats	Thermal hyperalgesia	TRP antagonism	[61]
	SB-366791, a TRPV1 antagonist	0.5 mg/kg, i.p.	Mice	Visceral nociception, mechanical hypersensitivity and heat hypersensitivity	TRPA1 antagonism	[55]
	Tabernaemontana catharinensis ethyl acetate fraction	100 mg/kg, p.o.	Mice	Mechanical allodynia	TRPA1 antagonism	[64]
Glutamate	Memantine	1–5 mg/kg	Rats	Mechanical hypersensitivity	Antagonism of NMDA receptor	[65]
	Valproate	200 mg/kg, i.p.	Rats	Mechanical allodynia	Suppressions HDAC2 upregulation, glutamate accumulation, and the corresponding changes in EAAT2/VGLUT/synaptophysin expression and HDAC2/YY1 interaction	[66]
PDE	Cilostazol	Diet containing 0.3% cilostazol	Mice	Mechanical hyperalgesia and Schwann cell dedifferentiation within the sciatic nerve	Differentiation of Schwann cells via a mechanism involving cAMP/Epac signaling	[67]
	Minoxidil	25–50 mg/kg, i.p.	Mice	Mechanical hyperalgesia, thermal sensitivity, and damages of sciatic nerve	Suppression of neuroinflammation (macrophage and microglia) recruitments and remodeling of intracellular calcium homeostasis in DRG	[68]
Cannabinoid receptor	Cannabidiol	1–20 mg/kg, i.p.	Mice	Mechanical sensitivity	N.A.	[69]
	Cannabidiol	2.5–25 mg/kg, i.p., p.o.	Mice	Mechanical allodynia	N.A.	[70]
	JZL184, a MAGL inhibitor	4–40 mg/kg, i.p.	Mice	Mechanical allodynia	N.A.	[49]
	KLS-13019	2.5–25 mg/kg, i.p.	Mice	Mechanical allodynia	N.A.	[70]
	MDA7, a CB₂ agonist	15 mg/kg, i.p.	Rats	Mechanical allodynia	Downregulations of IRF8, P2X4, CaMKIIα, p-CREB, FosB, BDNF, GluR1 and NR2B, and increase in the expression of K^+^-Cl^-^ cotransporter	[48]
	MJN110, a MAGL inhibitor	4–40 mg/kg, i.p.	Mice	Mechanical allodynia	Downregulations of monocyte chemoattractant protein-1 (MCP-1 and CCL2) and p-p38 MAPK in dorsal root ganglia as well as MCP-1 in the spinal dorsal horn	[49]
	URB597, a centrally penetrant FAAH inhibitor	1 mg/kg, i.p.	Mice	Mechanical hypersensitivity and cold hypersensitivity	Inhibition of FAAH, the major enzyme catalyzing the degradation of anandamide, an endocannabinoid, and other fatty acid amides	[71]
	URB937, a peripherally restricted FAAH inhibitor	1 mg/kg, i.p.	Mice	Mechanical hypersensitivity and cold hypersensitivity	Inhibition of FAAH, the major enzyme catalyzing the degradation of anandamide, an endocannabinoid, and other fatty acid amides	[71]
	β-caryophyllene, a CB2 agonist	25 mg/kg, p.o.	Mice	Mechanical allodynia	CB2-activation in the CNS and posterior inhibition of p38 MAPK/NF-κB activation and cytokine release	[56]
	Δ9-THC	2.5–20 mg/kg, i.p.	Mice	Mechanical sensitivity	N.A.	[69]
Opioid receptor	Morphine	3–6 mg/kg, p.o.	Mice	Mechanical allodynia	N.A.	[72]
	Oxycodone	24 mg/kg/day, p.o.	Mice	Mechanical allodynia	N.A.	[72]
Monoamines	SR-17018	1–48 mg/kg/day, p.o.	Mice	Mechanical allodynia	N.A.	[72]
	Bee venom acupuncture	1 mg/kg, s.c.	Rats	Mechanical hyperalgesia	Via spinal α₂-adrenergic receptor	[73]
	Bee venom acupuncture	0.25–2.5 mg/kg, i.p.	Mice	Cold allodynia and mechanical allodynia	Via the spinal noradrenergic and serotonergic mechanism	[74]
	Quetiapine	10–15 mg/kg, p.o.	Mice	Heat hyperalgesia, mechanical allodynia, and cold allodynia	Via α2-adrenoceptors	[75]
	Reboxetine	10 mg/kg, i.p.	Rats	Mechanical allodynia and cold hyperalgesia	α2-AR mediated antinociception at the spinal cord	[76]
	Venlafaxine	40–60 mg/kg, s.c.	Mice	Cold allodynia and mechanical allodynia	Via the spinal noradrenergic and serotonergic mechanism	[74]
Acetylcholine receptor	Nicotine	0.6–0.9 mg/kg, i.p. or 24 mg/kg, s.c.	Mice	Mechanical allodynia and density of IENF	Via α7 nicotinic acetylcholine receptor	[77]
	Pirenzepine	10 mg/kg, s.c.	Mice	Mechanical sensitivity and thermal sensitivity	Muscarinic ACh type 1 receptor (M1R) antagonism	[78]
	R-47, an α7 nAChR silent agonist	5–10 mg/kg, i.p.	Mice	Mechanical hypersensitivity, loss of IENF and morphological changes of microglia	N.A.	[79]
	α-Conotoxin RgIA4	80 μg/kg, s.c.	Rats	Mechanical allodynia	N.A.	[80]
cAMP/PKA	ESI-09, a Epac inhibitor	20 mg/kg, p.o.	Mice	Mechanical allodynia and number of IENF	Suppression of spinal cord astrocyte activation	[40]
PKC	HOE140, a kinin B2 antagonist	50 nmol/kg, i.p.	Mice	Mechanical hyperalgesia	inactivation of PKCε	[62]
	DALBK, a kinin B1 antagonist	150 nmol/kg, i.p.	Mice	Mechanical hyperalgesia	inactivation of PKCε	[62]
	Tamoxifen	30 mg/kg, p.o.	Mice	Mechanical allodynia cold allodynia	Inhibition of PKC/ERK pathway	[81]
MAPK	Duloxetine	30 mg/kg/day, i.p.	Mice	Mechanical hyperalgesia, thermal hyperalgesia, and loss of IENF	Decreases in NF-κB, p-p38, IL-6, and TNF-α in DRG	[39]
	Duloxetine	10–30 mg/kg, p.o.	Mice	Mechanical allodynia and cold allodynia	Inhibiting ERK1/2 phosphorylation in spinal cord	[82]
	Gabapentin	30–100 mg/kg, p.o.	Mice	Mechanical allodynia and cold allodynia	Inhibiting ERK1/2 phosphorylation in spinal cord	[82]
	Lacosamide	30 mg/kg, p.o.	Rats	Thermal hyperalgesia and cold allodynia	Upregulation of total antioxidant capacity and NGF, and downregulation of NF-kB p65, TNF-α, active caspase-3, Notch1 receptor, p-p38, and IL-6/p-JAK2/p-STAT3	[28]
	MJN110, a MAGL inhibitor	4–40 mg/kg, i.p.	Mice	Mechanical allodynia	Downregulations of MCP-1, CCL2 and p-p38 in DRG as well as MCP-1 in the spinal dorsal horn	[49]
	PD0325901	30 mg/kg, p.o.	Mice	Mechanical allodynia and cold allodynia	Inhibiting ERK1/2 phosphorylation in spinal cord	[82]
	Pregabalin	30 mg/kg, p.o.	Rats	Thermal hyperalgesia and cold allodynia	Upregulation of total antioxidant capacity and NGF, and downregulation of NF-kB p65, TNF-α, active caspase-3, Notch1 receptor, p-p38, and IL-6/p-JAK2/p-STAT3	[28]
	Siwei Jianbu decoction	5–10 g/kg, p.o.	Mice	Mechanical hyperalgesia and thermal nociception	Inhibiting the JNK, ERK1/2 phosphorylation, NF-κB, TNF-α, IL-1β, and IL-6	[53]
	Tamoxifen	30 mg/kg, p.o.	Mice	Mechanical allodynia cold allodynia	Inhibition of PKC/ERK pathway	[81]
	Trametinib	0.5 mg/kg	Mice	Mechanical and cold allodynia	Inhibition of the MEK/ERK pathway	[83]
	β-caryophyllene, a CB2 agonist	25 mg/kg, p.o.	Mice	Mechanical allodynia	Through CB2-activation in the CNS and posterior inhibition of p38 MAPK/NF-κB activation and cytokine release	[56]
OATP1B2	Nilotinib	100 mg/kg, p.o.	Mice	Mechanical allodynia	Inhibition of paclitaxel intake to neuron via OATP1B2 inhibition	[84]
mTOR	Rapamaycin	5 mg/kg, i.p.	Rats	Mechanical hypersensitivity and thermal hypersensitivity	Decreases of IL-1β, IL-6, TNF-α, substance P and CGRP in DRG.	[51]
Others	AM9053, a NAAA inhibitor	1–10 mg/kg, i.p.	Mice	Mechanical allodynia	N.A.	[85]
	Aucubin	15–50 mg/kg, i.p.	Mice	Mechanical allodynia	N.A.	[86]
	Aucubin	50 mg/kg, i.p.	Mice	Mechanical allodynia	Inhibition of ER stress in peripheral Schwann cells	[87]
	Bogijetong decoction, a herbal drug formulation	400 mg/kg, p.o.	Rats	Heat sensitivity	Improvement of morphological abnormalities in the sciatic nerve axons and DRG tissue	[88]
	DALBK, a kinin B1 antagonist	150 nmol/kg, i.p.	Mice	Mechanical allodynia	Antagonism of kinin B1 receptor	[89]
	FR173657, a kinin B2 antagonist	100 nmol/kg, i.p.	Mice	Mechanical allodynia	Antagonism of kinin B2 receptor	[89]
	*Gelsemium sempervirens*	1 mL, i.p.	Rats	Mechanical allodynia, mechanical hyperalgesia, cold allodynia, and density of IENF	N.A.	[90]
	HOE140, a kinin B2 antagonist	100 nmol/kg, i.p.	Mice	Mechanical allodynia	Antagonism of kinin B2 receptor	[89]
	Iridoids isolated from Viticis Fructus	15 mg/kg	Mice	Mechanical allodynia	N.A.	[91]
	*Lepidium meyenii*	0.5–10 mg/kg, p.o.	Rats	Cold hypersensitivity	N.A.	[92]
	Metformin	200 mg/kg, i.p.	Mice	Mechanical hypersensitivity	Activation of AMPK	[93]
	Narciclasine	1 mg/kg, p.o.	Mice	Mechanical hypersensitivity	Activation of AMPK	[93]
	Neoline	10 mg/kg/day, s.c.	Mice	Mechanical hyperalgesia	N.A.	[94]
	Nicotinamide riboside	200 mg/kg, p.o.	Rats	Mechanical hyperalgesia and cold hyperalgesia	N.A.	[95]
	NO-711, a GAT-1 inhibitor	3 mg/kg, i.p.	Mice	Thermal hyperalgesia and cold allodynia	Inhibition of GAT-1	[96]
	Processed aconite root	1 g/kg/day, s.c.	Mice	Mechanical hyperalgesia	N.A.	[94]
	Recombinant human soluble thrombomodulin	3–10 mg/kg, i.p.	Rats	Mechanical hyperalgesia	Inactivation of HMGB1	[97]
	Rikkunshito	0.3–1 mg/kg, p.o.	Mice	Mechanical hyperalgesia	Suppression of p-NF-κB in spinal cord	[98]
	Salicylidene salicylhydrazide	50–75 mg/kg, i.p.	Mice	Mechanical allodynia and cold allodynia	N.A.	[99]
	*Sargassum glaucescens* from the Persian Gulf	100–200 mg/kg, i.p.	Mice	Cold allodynia	N.A.	[100]
	SLAB51, a probiotic formulation	1.5 g (200 billion of bacteria) in 10 mL of drinking water	Mice	Mechanical allodynia and hyperalgesia	Increases in the expression of opioid and cannabinoid receptors in spinal cord, reduction in nerve fiber damage in the paws and modulation of the serum proinflammatory cytokines concentration	[101]
	SSR240612, a kinin B1 antagonist	150 nmol/kg, i.p.	Mice	Mechanical allodynia	Antagonism of kinin B1 receptor	[89]
	Staurosporine	0.1 mg/kg, i.p.	Mice	Mechanical allodynia	Inhibitory of PI3K signaling pathway	[102]
	Telmisartan	5–10 mg/kg, i.p.	Mice	Mechanical hyperalgesia and thermal hyperalgesia	Inhibition of CYP2J isoforms and reductions of EpOME in DRGs and plasma	[103]
	Terfenadine	1–2 mg/kg	Mice	Mechanical hyperalgesia	Inhibition of CYP2J isoforms	[103]
	Wortmannin	0.6 mg/kg, i.p.	Mice	Mechanical allodynia	Inhibitory of PI3K signaling pathway	[102]

Abbreviations: Ach, acetylcholine; AMPK, AMP-activated protein kinase; Apaf-1, apoptosis protease-activating factor 1; ATF-3, activating transcription factor 3; Bcl-2, B-cell lymphoma 2; Bcl-xL, B-cell lymphoma-extra-large; BDNF, brain derived neurotrophic factor; CaMKIIα, calmodulin-dependent protein kinase IIα; CCL2, C-C motif chemokine ligand 2; CCR2, C-C motif chemokine receptor 2; CGRP, calcitonin gene-related peptide; CREB, cAMP response element binding protein; CXCR, C-X-C motif chemokine receptor; CYP2J, Cytochrome P450 2J; DRG, dorsal root ganglia; EAAT2, excitatory amino acid transporter 2; Epac, exchange protein directly activated by cAMP; EpOME, epoxyoctadecamonoenoic acids; ER, endoplasmic reticulum; ERK, extracellular signal-regulated kinase; FAAH, fatty-acid amide hydrolase; FosB, FBJ murine osteosarcoma viral oncogene homolog B; GAT-1, gamma-aminobutyric acid (GABA) transporter 1; GFAP, glial fibrillary acidic protein; GluR1, glutamate ionotropic receptor AMPA type subunit 1; GSH, glutathione; HDAC2, histone deacetylase 2; HMGB1, high mobility group box 1; HO-1, heme oxygenase 1; i.p., intraperitoneal; i.v., intravenous; IENF, intra-epidermal nerve fibers; IL-10, interleukin-10; IL-1β, interleukin-1 beta; IL-6, interleukin-6; IL-8, interleukin-8; iNOS, inducible nitric oxide synthase; IRF8, interferon regulatory factor 8; JNK, c-Jun N-terminal kinase; MAGL, monoacylglycerol lipase; MAPK, mitogen-activated protein kinase; MCP-1, monocyte chemotactic protein 1; MDA, malondialdehyde; MEK, mitogen-activated protein kinase kinases; MPO, myeloperoxidase; NAAA, N-acylethanolamine-hydrolyzing acid amidase; nAChR, nicotinic acetylcholine receptor; NF-κB, nuclear factor kappa-B; NGF, nerve growth factor; NMDA, N-methyl-D-aspartate; NOX4, nicotinamide adenine dinucleotide phosphate (NADPH) oxidase 4; NQO1, NAD(P)H dehydrogenase [quinone] 1; NR2B, N-methyl D-aspartate (NMDA) receptor subtype 2B; Nrf2, nuclear factor-erythroid 2-related factor 2; OATP1B2, organic anion-transporting polypeptide 1b2; p.o., per os; p-Akt, phospho-protein kinase B; PARP, poly ADP-ribose polymerase; p-CREB, phospho-cAMP response element binding protein; p-FAK, phospho-fokal adhesion kinase; PGC-1α, peroxisome proliferatoractivated receptor γ coactivator-1; PI3K, phosphatidylinositol-3 kinase; p-JAK2, phospho-janus kinase 2; PKC, protein kinase C; p-NF-κB, phospho-nuclear factor kappa-B; p-p38, phospho-p38; PPAR-α, peroxisome proliferator-activated receptor-α; p-STAT3, phospho-signal transducer and activator of transcription 3; RAGE, receptor for advanced glycation endproducts; s.c., subcutaneous; SIRT1, sirtuin-1; SNAP, sensory nerve action potential; SNCV, sensory nerve conduction velocity; SOD, superoxide dismutase; TLR4, Toll-like receptor 4; TNF-α, tumor necrosis factor-α; TRP, transient receptor potential; TRPA1, transient receptor potential ankyrin 1; TRPV1, transient receptor potential vanilloid 1; TRPV4, transient receptor potential vanilloid 4; UCP2, uncoupling protein 2; VGLUT, vesicular glutamate transporter 3; YY1, Yin-Yang 1.

**Table 2:** The therapeutic drugs for paclitaxel-induced peripheral neuropathy in clinical experiments.

Investigational Drug	Dose (Preventive or Curative)	Chemotherapy	Study Design	Patient Number	Summary	References
Acetyl-L-carnitine	3000 mg daily, p.o. (preventive)	Taxanes	Randomized, double-blind, placebo-controlled, multicenter study	409	Significant reduction in NTX scores (worsening of peripheral neuropathy) >2 years	[111]
Amifostine	910 mg/m^2^, i.v., before the paclitaxel administration (preventive)	Carboplatin/paclitaxel	Randomized, controlled study	38	Significant improvements in paresthesia and sensory motor impairment.	[112]
	910 mg/m^2^, i.v., before the paclitaxel administration (preventive)	Paclitaxel	Randomized, controlled study	37	No significant difference in any of the measures of neurotoxicity.	[113]
Duloxetine	40 mg daily, p.o. (20 mg/day for the first week) (curative)	Oxaliplatin, paclitaxel, vincristine, or bortezomib	Randomized, open-label, crossover study (vs vitamin B12)	34	Significant improvements in numbness and pain	[114]
	60 mg/day, p.o., (30 mg/day for the first week) (curative)	Taxane or platinum	Randomized, double-blind, placebo-controlled, crossover study	231	In all patients, RRs (95% CI) of experiencing 30% and 50% pain reduction were 1.96 (1.15–3.35) and 2.43 (1.11–5.30), respectively In taxane-treated patients, RRs (95% CI) of experiencing 30% and 50% pain reduction were 0.97 (0.41–2.32) and 1.22 (0.35–4.18), respectively (not significant)	[115]
Gabapentin	900 mg daily, p.o., (preventive)	Paclitaxel	Randomized, double-blind, placebo-controlled study	40	Significant improvements in the incidence of grades 2–3 neuropathy and NCV changes	[116]
Glutamate	1500 mg daily, p.o., (preventive)	Paclitaxel	Randomized, double-blind, placebo-controlled study	43	No significant difference in the frequency of signs or symptoms between the two groups	[117]
Glutathione	1.5 g/m^2^, i.v., immediately before chemotherapy (preventive)	Carboplatin/ paclitaxel	Randomized, double-blind, placebo-controlled study	185	No significant differences in acute pain score and EORTC QLQ-CIPN20 scores compared to the placebo group	[118]
Minocycline	200 mg daily, p.o., (preventive)	Paclitaxel	Randomized, double-blind, placebo-controlled, multicenter study	47	Significant improvements in acute pain score No significant differences in sensory neuropathy score of the EORTC QLQ-CIPN20 compared to the placebo group	[119]
N-acetyl cysteine	1200 mg daily or twice daily, p.o., (preventive)	Paclitaxel	Randomized, controlled, open label study	75	Significant improvements in incidence of grades 2–3 neuropathy, mTNS, and QOL scores Significant increase in serum NGF and decrease in serum MDA	[120]
Omega-3 fatty acid	1920 mg daily, p.o., (preventive)	Paclitaxel or oxaliplatin	Meta-analysis	116 (two trials)	Significant improvements in the incidence of peripheral neuropathy and SNAP amplitudes	[121]
	1920 mg daily, p.o., (preventive)	Paclitaxel	Randomized, double-blind, placebo-controlled study	57	Significant improvements in neuropathy incidence	[122]
Oral nutritional supplement containing EPA	p.o., (preventive)	Paclitaxel or cisplatin/carboplatin	Randomized, controlled study	92	Significant improvement in neuropathy	[123]
PARP inhibitors (olaparib or veliparib)	N.A.	Paclitaxel	Meta-analysis	843 (five trials)	Did not reduce the risk of chemotherapy-induced peripheral neuropathy	[124]
Pregabalin	150 mg daily, p.o., (curative)	Paclitaxel or docetaxel	Randomized, double-blind, controlled study (vs duloxetine group)	82	Improvements in NCI-CTCAE grade and PNQ scores were more significant with pregabalin in comparison to duloxetine	[125]
	150 mg daily, p.o., (preventive)	Paclitaxel	Randomized, double-blind, placebo-controlled, multicenter study	46	No significant differences in acute pain score and EORTC QLQ-CIPN20 scores compared to the placebo group	[126]
Recombinant human LIF	2 or 4 µg/kg, s.c., (preventive)	Carboplatin/ paclitaxel	Randomized, double-blind, placebo-controlled study	117	No significant difference in CPNE or any of the individual neurologic testing variables	[127]
Vitamin E	600 mg daily, p.o., (preventive)	Paclitaxel	Randomized, controlled study	32	Significant improvements in the incidence of neuropathy and PNP scores	[128]
	600 mg daily, p.o., (preventive)	Cisplatin or paclitaxel	Randomized, controlled study	31	Significant improvements in incidence and neuropathy scores	[129]

Abbreviations: 95% CI, 95% confidence interval; CPNE, composite peripheral nerve electrophysiology; EORTC QLQ-CIPN20, European Organisation for Research and Treatment of Cancer, Quality of Life-Chemotherapy-Induced Peripheral Neuropathy 20; EPA, eicosapentaenoic acid; LIF, leukemia inhibitory factor; MDA, malondialdehyde; mTNS, modified total neuropathy score; NCI-CTCAE, National Cancer Institute-Common Terminology Criteria for Adverse Events; NCV, nerve conduction velocity; NGF, nerve growth factor; NTX score, neurotoxicity score; PARP, poly ADP-ribose polymerase; PNP score, peripheral neuropathy score; PNQ, patient neurotoxicity questionnaire; QOL, quality of life; RR, relative risk; SNAP, sensory nerve action potential.

## Data Availability

No new data were created or analyzed in this study. Data sharing is not applicable to this article.

## Abbreviations

95% CI	95% confidence interval
Ach	acetylcholine
AMPK	AMP-activated protein kinase
Apaf-1	apoptosis protease-activating factor 1
ATF-3	activating transcription factor 3
Bcl-2	B-cell lymphoma 2
Bcl-xL	B-cell lymphoma-extra large
BDNF	brain derived neurotrophic factor
CaMKIIα	calmodulin-dependent protein kinase IIα
CCL2	C-C motif chemokine ligand 2
CCR2	C-C motif chemokine receptor 2
CGRP	calcitonin gene-related peptide
CPNE	composite peripheral nerve electrophysiology
CREB	cAMP response element binding protein
CXCL	C-X-C motif chemokine ligand
CXCR	C-X-C motif chemokine receptor
CYP2J	Cytochrome P450 2J
DRG	dorsal root ganglia
EAAT2	excitatory amino acid transporter 2
EORTC QLQ-CIPN20	European Organisation for Research and Treatment of Cancer: Quality of Life-Chemotherapy-Induced Peripheral Neuropathy 20
EPA	eicosapentaenoic acid
Epac	exchange protein directly activated by cAMP
EpOME	epoxyoctadecamonoenoic acids
ER	endoplasmic reticulum
ERK	extracellular signal-regulated kinase
FAAH	fatty-acid amide hydrolase
FosB	FBJ murine osteosarcoma viral oncogene homolog B
GAT-1	gamma-aminobutyric acid (GABA) transporter 1
GFAP	glial fibrillary acidic protein
GluR1	glutamate ionotropic receptor AMPA type subunit 1
GSH	glutathione
HDAC2	histone deacetylase 2
HMGB1	high mobility group box 1
HO-1	heme oxygenase 1
i.p.	intraperitoneal
i.v.	intravenous
IENF	intra-epidermal nerve fibres
IL-10	interleukin-10
IL-1β	interleukin-1 beta
IL-6	interleukin-6
IL-8	interleukin-8
iNOS	inducible nitric oxide synthase
IRF8	interferon regulatory factor 8
JNK	c-Jun N-terminal kinase
LIF	leukaemia inhibitory factor
MAGL	monoacylglycerol lipase
MAPK	mitogen-activated protein kinase
MCP-1	monocyte chemotactic protein 1
MDA	malondialdehyde
MEK	mitogen-activated protein kinase kinases
MPO	myeloperoxidase
mTNS	modified total neuropathy score
mTOR	mammalian target of rapamycin
NAAA	N-acylethanolamine-hydrolyzing acid amidase
nAChR	nicotinic acetylcholine receptor
NCI-CTCAE	National Cancer Institute-Common Terminology Criteria for Adverse Events
NCV	nerve conduction velocity
NF-κB	nuclear factor kappa-B
NGF	nerve growth factor
NMDA	N-methyl-D-aspartate
NOX4	nicotinamide adenine dinucleotide phosphate (NADPH) oxidase 4
NQO1	NAD(P)H dehydrogenase [quinone] 1
NR2B	N-methyl D-aspartate (NMDA) receptor subtype 2B
Nrf2	nuclear factor-erythroid 2-related factor 2
NTX score	neurotoxicity score
OATP1B2	organic anion-transporting polypeptide 1b2
p.o.	per os
p-Akt	phospho-protein kinase B
PARP	poly ADP-ribose polymerase
p-CREB	phospho-cAMP response element binding protein
PDE	phosphodiesterase
p-FAK	phspho-fokal adhesion kinase
PGC-1α	peroxisome proliferatoractivated receptor γ coactivator-1
PI3K	phosphatidylinositol-3 kinase
PIPN	paclitaxel-induced peripheral neuropathy
p-JAK2	phospho-janus kinase 2
PKA	protein kinase A
PKC	protein kinase C
p-NF-κB	phospho-nuclear factor kappa-B
PNP score	peripheral neuropathy score
PNQ	patient neurotoxicity questionnaire
p-p38	phospho-p38
PPAR-α	peroxisome proliferator-activated receptor-α
p-STAT3	phospho-signal transducer and activator of transcription 3
QOL	quality of life
RAGE	receptor for advanced glycation endproducts
RR	relative risk
s.c.	subcutaneous
SIRT1	sirtuin-1
SNAP	sensory nerve action potential
SNCV	sensory nerve conduction velocity
SOD	superoxide dismutase
TLR4	Toll-like receptor 4
TNF-α	tumour necrosis factor-α
TRP	transient receptor potential
TRPA1	transient receptor potential ankyrin 1
TRPV1	transient receptor potential vanilloid 1
TRPV4	transient receptor potential vanilloid 4
UCP2	uncoupling protein 2
VGLUT	vesicular glutamate transporter 3
YY1	Yin-Yang 1
